# Effect of cinnamamides on atopic dermatitis through regulation of IL-4 in CD4^+^ cells

**DOI:** 10.1080/14756366.2019.1569647

**Published:** 2019-02-06

**Authors:** Eun-Ju Choi, Young Bae Ryu, Yujiao Tang, Bo Ram Kim, Woo Song Lee, Trishna Debnath, Meiqi Fan, Eun-Kyung Kim, Hyun-Su Lee

**Affiliations:** a Department of Physical Education, College of Education, Daegu Catholic University, Gyeongsan, Republic of Korea;; b Natural Product Material Research Center, Korea Research Institute of Bioscience and Biotechnology, Jeongeup, Republic of Korea;; c Division of Food Bioscience, College of Biomedical and Health Sciences, Konkuk University, Chungju, Republic of Korea;; d Changchun University of Science and Technology, Changchun, China;; e Department of Food Science and Biotechnology, Dongguk University, Goyang, Republic of Korea;; f College of Pharmacy, Keimyung University, Daegu, Republic of Korea

**Keywords:** Atopic dermatitis, cinnamamides, Th1/Th2 cytokines, IL-4, CD4+ T cells

## Abstract

This study aimed to evaluate the effects of cinnamamides on atopic dermatitis (AD) and the mechanisms underlying these effects. To this end, the actions of two cinnamamides, (*E*)-3-(4-hydroxyphenyl)-*N*-phenylethyl acrylamide (NCT) and *N*-*trans*-coumaroyltyramine (NCPA), were determined on AD by orally administering them to mice. Oral administration of the cinnamamides ameliorated the increase in epidermal and dermal thickness as well as mast cell infiltration. Cinnamamides suppressed serum immunoglobulin (Ig) levels and expression of T-helper (Th)1/Th2 cytokines. Moreover, cinnamamides suppressed interleukin (IL)-4, which plays a crucial role in preparing naïve clusters of differentiation (CD)4^+^ T cells, and decreased the cervical lymph node size and weight. Interestingly, in almost all cases, NCPA exhibited higher anti-AD activity compared to NCT. These results strongly indicate that NCPA may have potential as an anti-AD agent, and further mechanistic comparative studies of NCT and NCPA are required to determine the cause of differences in biological activity.

## Introduction

Atopic dermatitis (AD) is a complex incurable inflammatory skin disease associated with pruritus, dryness, erythema, weeping, and eczematous skin lesions^1^. AD usually presents during early infancy and childhood; however, it can start or may continue into middle age. AD is a multifaceted condition with precipitating factors including an abnormal immune system, genetic disorders, and environmental factors^2–5^. Distributed skin barrier functions lead to transepidermal water loss and T-helper 1 (Th1)- and Th2-mediated immunological responses. Th2 cell-mediated changes in interleukin-4 (IL-4), IL-5, and IL-13 expressions have been reported in the acute phase of AD, whereas in the chromic stages, Th1 cell-mediated AD lesions were found^6^^,^^7^. In addition, tumour necrosis factor (TNF)-α and interferon (IFN)-γ are involved in acute and chronic AD^8^^,^^9^.

Moisturisers and topical steroids are used for AD treatment to maintain the moisture level of the skin and reduce inflammation, respectively. However, long-term use of topical steroids or short, repeated courses of stronger steroids are associated with adverse events^10^. Therefore, numerous research studies have recently focused on developing natural anti-inflammatory agents with low incidence of side effects^11^.

Biochemical properties of polyphenolic secondary plant metabolites such as esters of cinnamic acids (e.g. caffeic, ferulic, and *p*-, and *o*-coumaric acids) have attracted considerable attention in medicine^12^. Cinnamic acid derivatives have low toxicity and show anti-allergic, anti-inflammatory, free radical scavenging, antitumor, and antimicrobial activities^13–15^. Cinnamamides are N-substituted amides of cinnamic acids and, as a scaffold of interest to medicinal chemistry, have been incorporated into synthetic compounds with antimicrobial^16^, neuroprotective^17^, and anti-inflammatory properties^18^.

This study evaluated and compared the inhibitory effect of synthesised cinnamamides, (2*E*)-3–(4-hydroxyphenyl)-*N*-(2-phenylethyl) acrylamide (NCPA) and *N*-*p*-*trans*-coumaroyltyramine (NCT) on AD induced by *Dermatophagoides farinae* extract (DFE, house dust mite extract) and 2,4-dinitrochlorobenzene (DNCB) in BALB/c mice. Specifically, it investigated the effect of the compounds on epidermal and dermal ear thickness, mast cell infiltration, serum immunoglobulin (Ig) levels, and mRNA expression of Th1/Th2 cytokines in ear skin tissue. In addition, the expression of IL-4 in clusters of differentiation (CD)4^+^ T cells, which play central roles in the function of the immune system^19^, in draining lymph nodes (dLN), and the size and weight of cervical lymph nodes were determined.

## Materials and methods

### Materials and cell culture

The TRIzol reagent for RNA extraction was obtained from Invitrogen (Carlsbad, CA, USA), and the primary antibodies and peroxidase-conjugated secondary antibodies were purchased from Santa Cruz Biotechnology Inc. (Santa Cruz, CA, USA). Jurkat cells (human T lymphocytes) were obtained from the ATCC (Manassas, VA, USA) and cultured in RPMI-1640 Medium (Sigma-Aldrich, St. Louis, MO, USA) supplemented with 10% fetal bovine serum (Gibco, Thermo Fisher Scientific, Waltham, MA, USA) at 37 °C in a 5% CO_2_ atmosphere. All other reagents were of the highest grade commercially available at the time of the study.

### General procedure and characterisation of amide derivatives

*p*-Coumaric acid (1.0 g, 61.09 mmol, 1.0 equiv.) was dissolved in dimethylformamide (20 mL) and stirred at 0 °C for 5 min. Triethylamine (0.85 mL, 6.09 mmol, 1.0 equiv.) was added drop-wise and stirred for 5 min before the corresponding amines (6.09 mmol, 1.0 equiv.) were added, followed by a solution of benzotriazol-1-yloxy-tris-(dimethylamino)-phosphonium hexafluorophosphate (BOP) in dichloromethane (CH_2_Cl_2_, 20 mL). The mixture was stirred at 0 °C for 30 min and then at room temperature overnight. The reaction mixture was evaporated under reduced pressure; ethyl acetate (30 mL) and water (H_2_O, 20 mL) were added, and the organic layer was washed with 1 N hydrochloric acid (HCl, aq., 20 mL) and sodium bicarbonate (NaHCO_3_, aq., saturated, 20 mL). The organic layer was separated and dried over anhydrous magnesium sulfate (MgSO_4_). The solvent was evaporated under reduced pressure, and the residue was further purified using an open-bed silica gel column (4 × 30 cm, ethyl acetate:*n*-hexane:methanol, 6:2:1, v/v/v). Fractions containing the product were combined and evaporated under reduced pressure to obtain the corresponding amides.

### Nct

The synthesised NCT had the following characteristics: yellowish powder; melting point (mp), 143–146 °C; EIMS, *m*/*z* 267 [M]^+^; HREIMS, *m*/*z* 267.1259 (calcd for C_17_H_17_NO_2_ 267.1259); IR (KBr) 3423, 3140, 3015, 2951, 1655 cm^−1^; ^1^H-NMR (CD_3_OD, 300 MHz) δ: 7.48 (1H, d, *J* = 15.7 Hz), 7.42 (2H, d, *J* = 8.6 Hz), 7.24 (2H, m), 7.04 (2H, m), 6.99 (1H, br), 6.81 (2H, d, *J* = 8.6 Hz), 6.41 (1H, d, *J* = 15.7 Hz), 3.51 (2H, t, *J* = 7.3 Hz), 2.84 (2H, t, *J* = 7.3 Hz). ^13^C-NMR (75 MHz) δ: 167.8 (C-9), 159.1 (C-4), 140.4 (C-7), 135.1 (C-1′), 130.1 (C-3′, C-5′), 129.1 (C-2′, C-6′), 126.2 (C-4′), 116.9 (C-8), 115.3 (C-3, C-5), 114.7 (C-2, C-6), 114.5 (C-1), 40.7 (C-8′), 34.3 (C-7′). NCT was dissolved in DMSO for *in vitro* and *in vivo* experiments.

### Ncpa

The synthesised NCPA had the following characteristics: white powder; melting point (mp), 255–257 °C; electron impact mass spectrometry (EIMS), *m*/*z* 283 [M]^+^; high-resolution (HR) EIMS, *m*/*z* 283.1209 (calcd for C_17_H_17_O_3_ 283.1208); IR (KBr) 3432, 3300, 3175, 3023, 2940, 1660 cm^−1^; proton nuclear magnetic resonance (^1^H-NMR, CD_3_OD, 500 MHz) δ: 7.36 (1H, d, *J* = 15.7 Hz), 7.30 (2H, d, *J* = 8.6 Hz), 6.96 (2H, d, *J* = 8.4 Hz), 6.69 (2H, d, *J* = 8.6 Hz), 6.62 (2H, d, *J* = 8.4 Hz) 6.30 (1H, d, *J* = 15.7 Hz), 3.37 (2H, t, *J* = 7.4 Hz), 2.66 (2H, t, *J* = 7.5 Hz). ^13^C-NMR (125 MHz) δ: 169.7 (C-9), 160.9 (C-4), 157.4 (C-4′), 142.2 (C-7), 131.8 (C-1′), 131.1(C-2, C-6), 130.9 (C-2′, C-6′), 128.2 (C-1), 118.9 (C-8), 117.1 (C-3, C-5), 116.7 (C-3′, C-5′), 42.9 (C-8′), 36.2 (C-7′). NCPA was dissolved in DMSO for *in vitro* and *in vivo* experiments.

### Animals

Eight-week-old female BALB/c mice were purchased from Samtako (Osan, Republic of Korea) and housed under specific-pathogen-free conditions. All the experiments were approved by the Institutional Animal Care and Use Committee (IACUC) of Konkuk University, Korea (Protocol no. KU14012).

### Induction of AD lesions in the ear

**Figure 1.**
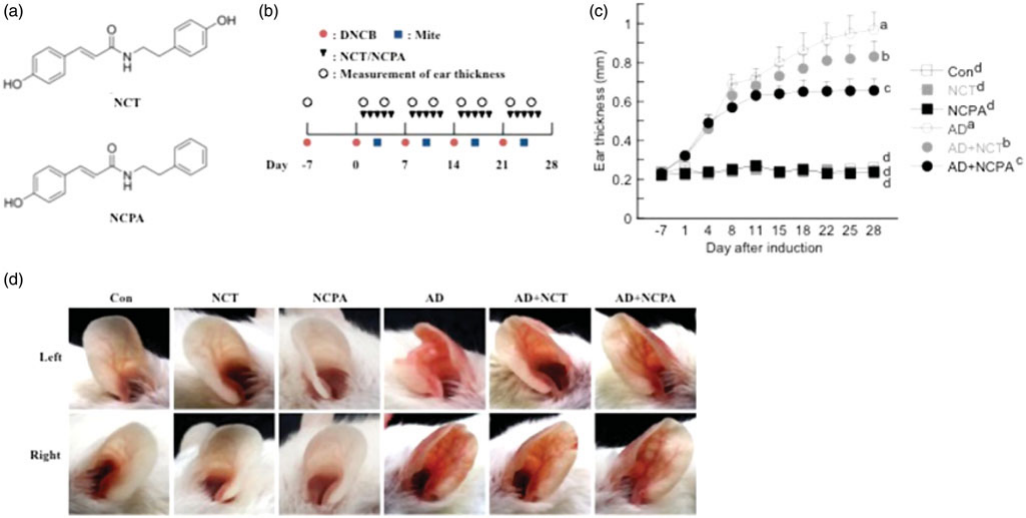
(a) Structures of NCT and NCPA. (b) Schematic depiction of the induction and NCT and NCPA treatment of atopic dermatitis (AD). (c) Ear thickness during the course of AD. (d) Representative photographs of mouse ears from each group at 28 days after AD induction. Means denoted with different letters (a–d) within a graph are significantly different from each other at *p* < 0.05.

AD was induced in the mice by repeated local exposure of the ears to DFE and DNCB, as previously described^20^. For the induction of AD, the mice were divided into six groups (control, AD-only, NCT-only, NCPA-only, AD + NCT, and AD + NCPA). Structures of NCT and NCPA and experimental design are shown in Figure 1(a,b), respectively. To induce AD, the surfaces of both earlobes were stripped five times using surgical tape (Nichiban, Tokyo, Japan), and 20 µL 1% DNCB was applied to each ear, followed 4 days later by 20 µL of DFE (10 mg/mL). DFE and DNCB treatments were administered alternately once per week for 4 weeks. The animals in NCT-only, NCPA-only, AD + NCT, and AD + NCPA groups were orally administrated NCT or NCPA (50 mg/kg/day) throughout the 4-week AD induction period.

The ear thickness was measured 24 h after DNCB or DFE applications using a dial thickness gauge (ID-C1012XBS, Mitutoyo Co., Kawasaki, Japan). On days 14 and 28, blood samples were collected by orbital puncture; plasma samples were prepared, and stored at –70 °C for further analysis. After blood collection on day 28, mice ears were removed and histopathologically analysed. Serum IgE and IgG2a levels were measured on days 14 and 28 after the first induction using an IgE enzyme-linked immunoassay kit (Bethyl Laboratories, Inc., Montgomery, TX, USA) according to the manufacturer’s instructions.

### Histological observations

The excised mouse ears were fixed in 4% paraformaldehyde for 16 h and embedded in paraffin, and thin (6 µm) sections were cut and stained with hematoxylin and eosin (H&E). The thicknesses of the epidermis and dermis were measured under a microscope. For determining mast cell infiltration, skin sections were stained with toluidine blue, and the mast cells were counted in five randomly chosen fields of view.

### CD4^+^ T-cell preparation

Naïve BALB/c mice were sacrificed and CD4^+^ T cells were isolated from the LNs by magnetic-activated cell sorting (MACS) separation (Miltenyi Biotec, Bergisch Gladbach, Germany).

Jurkat cells were stimulated with phorbol myristate acetate (PMA)/calcium ionophore A23187 for 24 h. The supernatants were collected, and IL-2 mRNA levels were determined.

### Analysis of mRNA expression

For the reverse transcription-polymerase chain reaction (RT-PCR), the total cellular RNA was isolated from the ear tissue and the CD4^+^ T cell in draining lymph nodes (dLNs) and non-dLNs using TRIzol according to the manufacturer’s protocol [20]. The first-strand complementary DNA (cDNA) was synthesised using Superscript II reverse transcriptase (Invitrogen). The conditions for RT-PCR were similar to those employed in previously described studies^21^.

Quantitative real-time PCR (qPCR) was performed in triplicate using 12.5-μL SYBR Premix Ex Taq (Takara, Japan) and 2-μL cDNA as a template in 25-μL final volume. The primers used for qPCR were as follows: mouse IL-2 forward primer: ATG TAC AGC ATG CAG CTC GCA TCC TGT GTC A, reverse primer: AGT CAA ATC CAG AAC ATG CCG CAG ACG TCC A; mouse TNF-α forward primer: AAG CCT GTA GCC CAC GTC GT, reverse primer: GGC ACC ACT AGT TGG TTG TC; IL-4 forward primer: ACA GGA GAA GGG ACG CCA T, reverse primer: GAA GCC GTA CAG ACG AGC TC; mouse IL-5 forward primer: GAA GTG TGG CGA GGA GAG AC, reverse primer: GCA CAG TTT TGT GGG GTT TT; mouse IL-13 forward primer: GCA ACA TCA ACA GGA CCA GA, reverse primer: GTC AGG GAA TCC AGG GCT AC; and mouse glyceraldehyde 3-phosphate dehydrogenase (GAPDH) forward primer: GCA CAG TCA AGG CCG AGA AT, reverse primer: GCC TTC TCC ATG GTG GTG AA. The PCR amplification (40 cycles) was preceded by incubation of the mixture for 15 min at 95 °C. The following schedule was followed: denaturation for 30 s at 95 °C, annealing at a transitional temperature range from 58 to 62 °C with an increase of 0.5 °C per cycle, and extension for 30 s at 72 °C with fluorescence detection at 72 °C after each cycle. After the final cycle, melting profiles of all samples were analysed within the range of 65 to 95 °C with continuous fluorescence detection. The target gene mRNA levels were normalised to glyceraldehyde 3-phosphate dehydrogenase (GAPDH) levels using the following formula:

$$\text{relative }\text{mRNA }\text{expression}=2^{-(}{}^{\Delta \text{Ct }\text{of }\text{target }\text{gene}-\Delta \text{ Ct }\text{ of }\text{GAPDH})},$$

where *C*_t_ is the threshold cycle value. Expression levels of the analysed genes were normalised to GAPDH for each sample and presented as relative mRNA levels.

### Statistical analysis

Statistical analysis was carried out using SAS 9.1.3 software (SAS Institute, Cary, NC, USA). Differences between groups were analysed using a one-way analysis of variance (ANOVA) followed by Dunnett’s multiple range test. Data are expressed as means ± standard deviations (SDs) of comparative fold differences and are representative of three independent experiments. The threshold for significance was set at *p* < 0.05.

## Results and discussion

### Effect of NCT and NCPA on ear thickness, histopathological observations, and serum Ig levels

**Figure 2.**
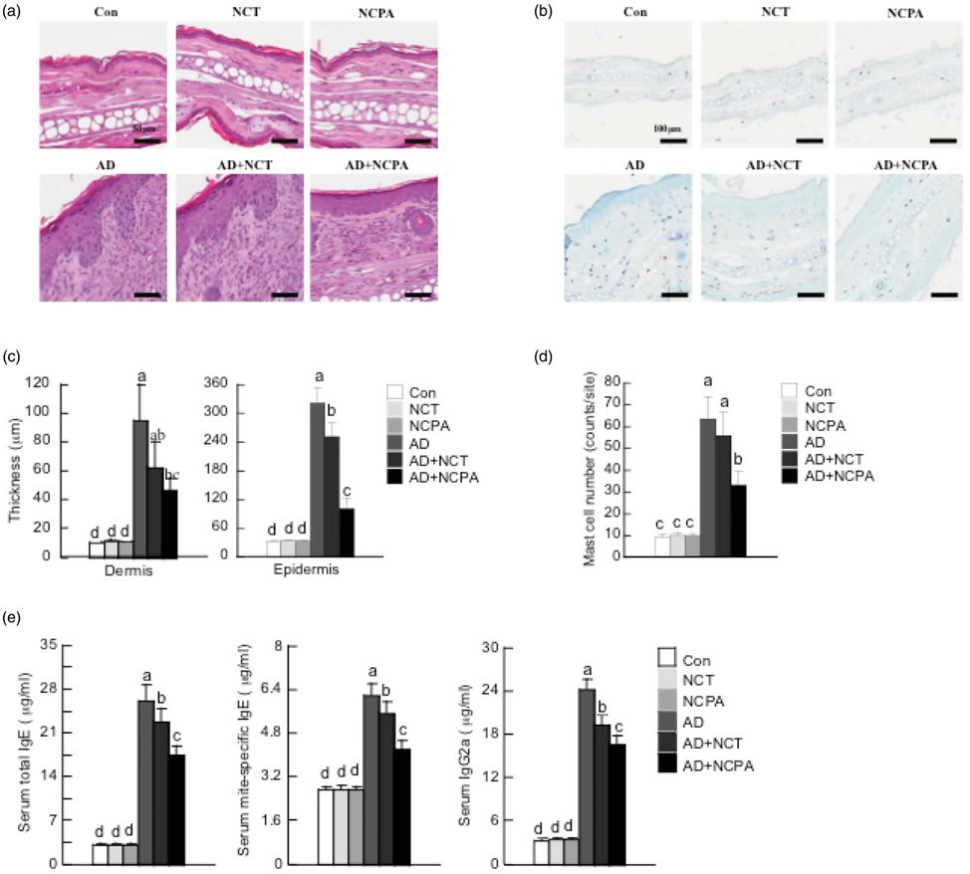
Histopathological and serum analysis to assess the effect of NCT and NCPA on atopic dermatitis (AD). Representative photomicrographs of ear skin tissue sections stained with hematoxylin and eosin or toluidine blue. (a,b) Epidermal and dermal thicknesses were measured using microphotographs of hematoxylin- and eosin-stained-tissue. (c,d) The number of infiltrated mast cells was determined based on toluidine blue staining. (e) Blood samples were collected by orbital puncture at day 28. Plasma total IgE, mite-specific IgE, and IgG2a levels were quantified by enzyme-linked immunosorbent assay. Data are presented as the mean ± SD of triplicate experiments. Means with different letters (a–d) within each graph are significantly different from each other at *p* < 0.05. AD was induced by DFE and DNCB treatment. The pictures shown are representative of each group (*n* = 3–6). The original magnification was 100 × CON. CON: control; AD: atopic dermatitis.

Ear thickness and AD lesions significantly increased following the application of DFE and DNCB. Oral administration of NCT and NCPA suppressed the increase in dermal and epidermal thickness (Figures 1(c,d) and 2(a,b)). In addition, NCT and NCPA-treated groups showed a significant reduction in the number of infiltrated mast cells compared with the AD-only group (Figure 2(c,d)). Moreover, AD mice exhibited higher IgE and IgG2a levels compared to control. On the contrary, NCT and NCPA-treated mice showed significantly reduced total and DFE-specific IgE and IgG2a levels compared with the AD-only group (Figure 2(e)).

### Effect of NCT and NCPA on inflammatory stress in stimulated T cells

**Figure 3.**
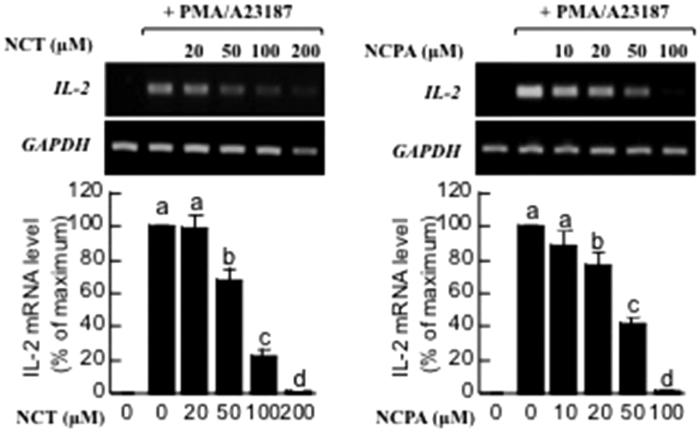
Effects of NCT and NCPA on the expression of IL-2 in Jurkat T lymphocytes. The cells were stimulated with PMA/A23187 for 24 h. The supernatants were collected, and IL-2 levels were measured using RT-PCR. Means denoted with different letters (a–d) within each graph are significantly different from each other at *p* < 0.05. PMA: phorbol myristate acetate.

Though the molecular basis of the process remains unclear, T-cell infiltration of the skin in AD has been well documented^22^. Therefore, the effects of cinnamamides on T cells were examined using Jurkat cells. Pretreatment with NCT and NCPA reduced IL-2 mRNA levels of Jurkat cells (Figure 3).

### Effect of NCT and NCPA on the expression of various pathogenic cytokines in vivo

**Figure 4.**
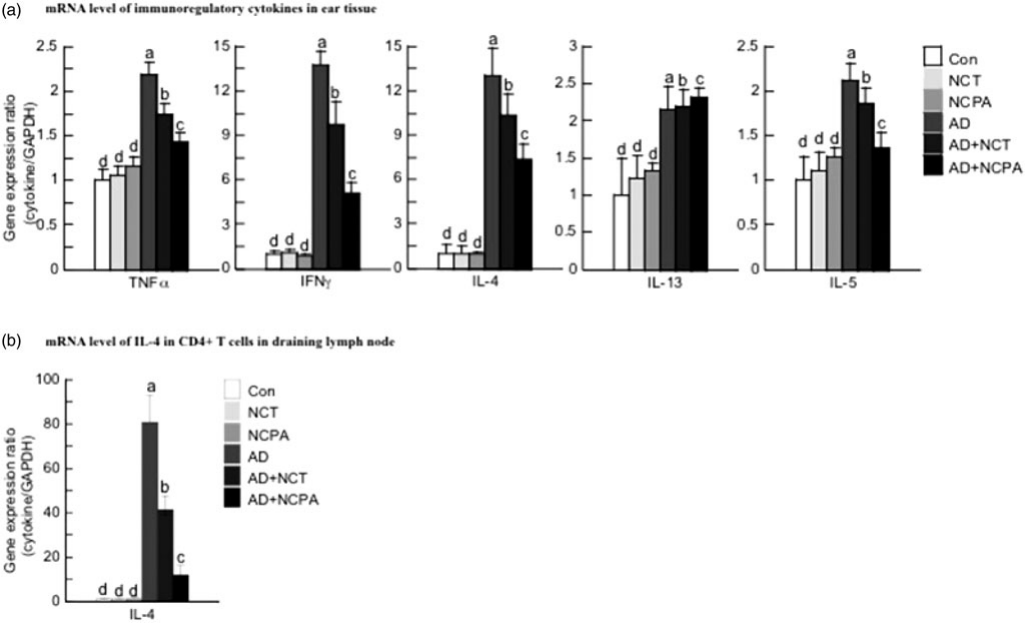
Effect of NCT and NCPA on the expression of immunoregulatory cytokines in the ear skin tissues (a) and CD4^+^ T cells in draining lymph nodes (b). The ear skin tissues were excised on day 28 and total RNA was isolated. Quantitative real-time polymerase chain reaction was performed as described in the Methods. Data are presented as the mean ± SD of triplicate determinations. *indicates significant difference from the value of the DFE/DNCB-treated mice at *p* < 0.05. AD was induced by DFE and DNCB treatment. DNCB: 2,4-dinitrochlorobenzene; DFE: *Dermatophagoides farina* extract; AD: atopic dermatitis. Means denoted with different letters (a–d) within each graph are significantly different from each other at *p* < 0.05.

The mRNA levels of the evaluated cytokines were upregulated in ear tissues of AD mice. Treatment with NCPA or NCT reduced the expression of Th1-related cytokines, IFN-γ and TNF-α, and Th2-related cytokines, IL-4, IL-5, and IL-13, in mouse ear tissues (Figure 4(a)). NCPA was significantly more effective than NCT in reducing mRNA expression (*p* < 0.05). In addition, as shown in Figure 4(b), NCT and NCPA suppressed IL-4 mRNA expression in CD4^+^ T cells in dLNs.

### Effect of NCT and NCPA on the morphology of the cervical lymph nodes cytokines in vivo

**Figure 5.**
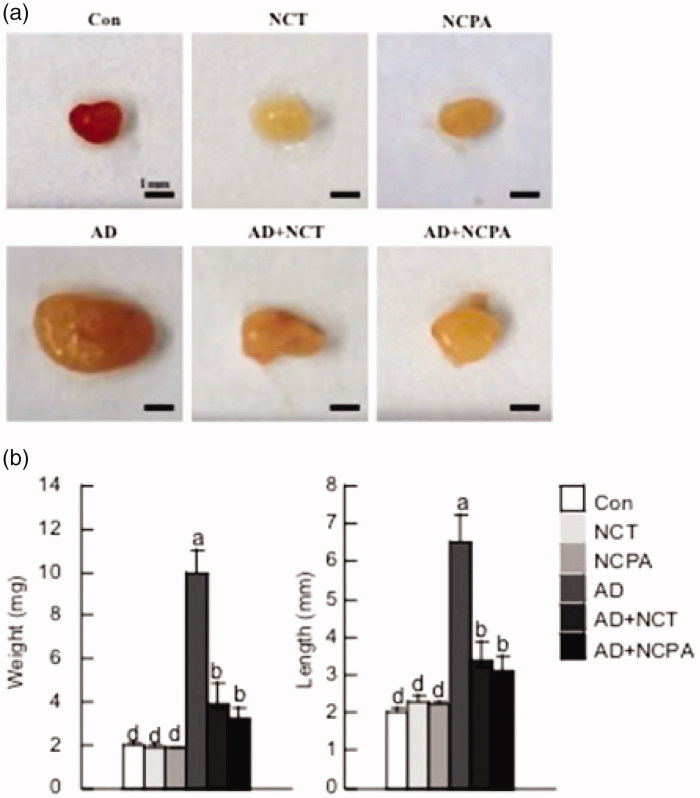
Representative photographs showing the size (a) and weight and length (b) of the cervical lymph nodes of the six mouse groups 28 days after AD induction. The data are represented as mean ± SD. Means denoted with different letters (a–d) within each graph are significantly different from each other at *p* < 0.05.

As AD often progresses as a systemic disease^23^, whether oral administration of NCT and NCPA affected systemic immune responses was examined. AD mice had larger and heavier cervical lymph nodes compared to the untreated control group. Oral administration of NCT and NCPA to AD mice significantly lowered the cervical lymph nodes weight and size compared to the AD-only group (Figure 5(a,b)).

Cinnamamides have a wide range of therapeutic effects, acting as immunomodulatory, anti-allergic, antitumor, and anti-infective agents^13^. In this study, two cinnamamides, NCT, and NCPA, were synthesised, and their therapeutic effects on AD, which was induced in BALB/c mice by alternately applying DFE and DNCB on both earlobes for 4 weeks, were comparatively evaluated. Oral administration of NCT and NCPA significantly suppressed ear thickness and AD lesions compared with the AD-only group, with NCPA showing a significantly higher activity.

Mast cells play a vital role in inflammation and, upon activation, release potent inflammatory mediators, including histamine and cytokines. AD-induced mouse ears were histologically analysed to confirm mast cell infiltration. Excised ear tissues from each group were stained with toluidine blue and microscopically examined. NCT- and NCPA-treated groups showed an inhibitory effect on the number of infiltrated mast cells compared with the AD-only group. In addition, NCPA treatment was more effective than NCT.

An increase in IgE levels is indicative of AD progression^24^, as AD is an immune disorder accompanied by increased serum IgE levels^25^^,^^26^. In addition, as chronic AD is frequently associated with high levels of IgG antibodies^27^, serum IgG2a levels were measured. IgE (total and DFE-specific) and IgG2a were measured for each treatment group. As expected, alternately applying DFE and DNCB increased IgE and IgG2a levels compared to control. However, NCT and NCPA significantly reduced total and DFE-specific IgE and IgG2a levels compared with AD-only group mice. Interestingly, NCPA was also more effective than NCT.

Th1/Th2 cell-mediated lesions are critical to AD progression, making the suppression of T-cell activity the optimal therapeutic approach for AD. In this study, treatment with NCT and NCPA reduced IL-2 mRNA levels in activated T cells.

In order to examine whether NCT and NCPA exerted their effects via the Th1 or Th2 response and elucidate the underlying mechanisms, the mRNA expression levels of AD-related cytokines in ear tissues were measured using real-time PCR. NCPA and NCT suppressed the expression of Th1- and Th2-related cytokines in mouse ear tissues. Similar with previous results, NCPA was significantly more effective compared to NCT (*p* < 0.05).

In addition, Matsui et al. reported that cinnamamides inhibited histamine release and TNF-α production in stimulated rat basophilic leukaemia cells (RBL-2H3)^26^. Acute AD lesions are characterised by an inflammatory cascade caused by the release of soluble factors including Th2-derived cytokines IL-4, IL-5, and IL-13 and increased serum Ig. As IL-4 plays an important role in preparing naïve CD4^+^ T cells for conversion into Th2 cells^28^; thus, the present study investigated whether NCT and NCPA regulate this process *in vitro*, by examining mRNA expression of IL-4 in CD4^+^ T cells in dLNs. In this study, NCT and NCPA reduced IL-4 mRNA expression, with NCPA displaying higher activity. Predominantly Th2-mediated response occurs in the acute phase of AD, whereas chronic AD is characterised by a predominantly Th1-mediated response. Our results suggest that NCT and NCPA inhibit the expression of Th1 and Th2 cytokines, indicating the potential therapeutic use of these cinnamamides in acute and chronic stages of AD.

From the present results, NCPA exhibited a higher effect compared to NCT. NCPA is an amphiphilic compound containing hydrophilic 4-hydroxyl amide groups and a hydrophobic phenyl group. Therefore, we infer that the amphiphilicity contributes to the observed results. Some amphiphilc compounds directly permeate cell membranes^29^. Therefore, we hypothesise that NCPA is effectively distributed between the lipophilic and hydrophilic compartments in AD.

## Conclusions

The present study examined the anti-AD potential of the synthesised cinnamamides NCT and NCPA following oral administration, using a mouse model of AD. Oral administration of NCT and NCPA each significantly decreased the severity of AD including histopathological features, production of Ig, and expression of Th1/Th2 cytokines in DFE/DNCB-induced BALB/c mice. Taken together, our results demonstrate that NCT and NCPA effectively suppress the AD-like phenotype by regulating the inflammatory response without inducing any significant effects on the normal immune responses. Furthermore, NCPA exhibited higher anti-AD activity compared to NCT.

Therefore, we propose NCPA as a promising candidate for development of alternative AD treatments. We suggest further mechanistic comparative studies of NCT and NCPA to establish the causes of the differences in biological activity of these compounds.
